# Tactile Learning in Cancer Awareness: Evaluating Prosthetic Models Versus Posters in Male Reproductive Health Education

**DOI:** 10.7759/cureus.99166

**Published:** 2025-12-13

**Authors:** Laith Fada, Emmanuel Garrido-Cortes, Matthew Martinez, Osama Fattouh, Rahul Garg, James R Nolin

**Affiliations:** 1 College of Osteopathic Medicine, Alabama College of Osteopathic Medicine, Dothan, USA; 2 Department of Research, Alabama College of Osteopathic Medicine, Dothan, USA; 3 Department of Simulation, Alabama College of Osteopathic Medicine, Dothan, USA

**Keywords:** medical education research, prostate cancer awareness, prosthetics, public health education, testicular cancer awareness

## Abstract

Prostate and testicular cancers represent major health concerns for men, yet public knowledge, awareness of how to perform self-examinations, and comfort discussing male reproductive health are often poor, with stigma further limiting early detection and prevention. This study compared the effectiveness of two educational modalities, poster-based instruction and prosthetic model-based instruction, in an effort to address these shortcomings among adult participants. Using a pre-, post-, and two-week follow-up survey design, we measured knowledge gains, retention, and comfort discussing male cancers with healthcare providers across different education levels and age groups. Prosthetic-based instruction consistently outperformed posters, producing greater immediate knowledge gains, stronger retention at two weeks, and larger improvements in confidence, particularly among participants with lower educational levels. By engaging learners through multisensory teaching methods and experiential practice, prosthetic models hold promise for reducing educational disparities, improving early-detection behaviors, and ultimately decreasing mortality from male reproductive cancers.

## Introduction

Prostate and testicular cancers are among the most prevalent male-specific malignancies worldwide, with prostate cancer being the second leading cause of cancer-related death in men and testicular cancer representing the most common malignancy in young adult males aged 15-35 [1,2]. Despite the availability of effective treatments, especially when diagnosed early, thousands of lives are lost each year due to delayed detection, lack of awareness, and stigma surrounding male reproductive health [3].

According to the American Cancer Society, over 34,000 men die annually from prostate cancer in the United States alone, and although testicular cancer has a high survival rate, delayed diagnosis leads to unnecessary morbidity, long-term treatment complications, and even death [1]. These figures underscore the vital importance of early detection, routine screening, and public education in reducing preventable deaths and improving quality of life.

Education regarding cancer prevention is one of the most important components, but the most effective mode of delivering this information remains uncertain [4]. Traditional educational tools such as posters are widely used due to their simplicity and scalability. Yet, they may lack the tactile and immersive engagement necessary to foster deeper understanding and behavioral change [4]. In contrast, prosthetic teaching tools, which allow individuals to physically interact with models representing the prostate or testicles, may offer superior engagement and retention among men with varying levels of education, especially in promoting self-examination skills and overcoming discomfort associated with male cancer topics [5].

This research aims to assess and compare the impact of poster-based versus prosthetic-based educational interventions on knowledge, retention, confidence, and comfort regarding prostate and testicular cancer. By evaluating these methods, we hope to inform future public health strategies and optimize cancer education efforts. Even modest improvements in awareness could lead to thousands of earlier diagnoses, resulting in significant reductions in mortality and lifelong health complications. Ultimately, this study contributes to the broader goal of empowering men with the knowledge and tools to take charge of their health, saving lives through simple, effective education.

## Materials and methods

This study utilized a quasi-experimental, pre-post intervention design to assess the effectiveness of two educational modalities, posters and life-sized prosthetic teaching models, in improving men’s knowledge, self-efficacy, and comfort with discussing male health topics related to prostate and testicular cancer. The primary outcome was the comparative impact of each teaching tool on participants’ learning outcomes, while secondary analyses explored the influence of demographic factors such as formal education level and age on the impact of each tool.

All men 18+ were allowed to participate, and we received a total of 76 valid participants, excluding all those who failed to reply and follow up. They were randomized via computer-generated sequence into four stratified study groups by cancer type (prostate vs. testicular) and educational delivery tool (poster vs. prosthetic). The groups were as follows: Group A (prostate and poster), Group B (prostate and prosthetic), Group C (testicular cancer and poster), and Group D (testicular cancer and prosthetic). Each group received standardized educational content covering epidemiology, risk factors, symptoms, screening guidelines, and self-exam techniques, delivered by trained medical students after a preparatory session to ensure uniformity in instruction. The preparatory session was conducted to ensure consistency and standardization of all informational content delivered across study groups. Participants assigned to either the testicular cancer or prostate cancer arms received the same key educational points within their respective topic areas, including information on disease symptoms, screening recommendations, and self-examination techniques. The only distinction between groups was the modality through which this information was presented, either by reviewing a visually formatted poster or by engaging in a hands-on demonstration using a prosthetic model. This approach ensured that any observed differences in participant outcomes could be attributed to the mode of information delivery rather than to differences in the content itself. Each intervention lasts approximately five minutes and is followed by participant Q&A.

The assessments included three surveys: pre-intervention baseline, immediate post-intervention, and a two-week follow-up administered by email. The surveys measured knowledge (via a 0-11 scale, seven multiple-choice and true/false items tailored for prostate or testicular cancer) and confidence in self-examination and comfort discussing cancer issues with medical professionals via a 1-4 Likert scale [6]. A shortened version of the Prostate Cancer Knowledge Scale (PrCA-KS) [7] and the Testicular Cancer Awareness and Knowledge Questionnaire [8] was developed to create a brief, targeted 11-item tool for assessing cancer-related knowledge in males. Items were selected from validated domains of each instrument, focusing on core concepts such as risk factors, early signs and symptoms, and treatment (Appendices). Both instruments are free to use in academic research. The baseline survey also measured demographic variables of education (high school or less, in college, bachelor’s, postgraduate). An IRB was obtained prior to data collection. Every participant signed a consent form. All identifying features have been removed.

Assessment tools and procedures

Evaluation of the intervention was carried out using pre-, post-, and two-week follow-up surveys designed to measure the effectiveness of each teaching tool employed. Immediately before the educational session, all participants completed a baseline pre-survey. This survey was composed of three domains: (1) knowledge regarding either prostate or testicular cancer (depending on group assignment), including risk factors, signs and symptoms, and appropriate self-examination or screening practices; (2) self-reported comfort in discussing sensitive male health topics with physicians; and (3) confidence in their ability to correctly perform a cancer-specific self-examination. The knowledge component was assessed through multiple-choice and true/false questions, while comfort and confidence were captured using a four-point Likert scale ranging from 1 (very uncomfortable and not confident at all) to 4 (very comfortable and very confident).

Immediately after completing the educational intervention, participants were administered a post-survey identical in structure and content to the pre-survey. This allowed for direct comparisons of knowledge acquisition depending on the education tool used, as well as immediate changes in confidence and comfort levels attributable to it. To evaluate retention and the durability of knowledge and application, the same survey was readministered two weeks after the intervention via either email or telephone, depending on participant preference. The follow-up survey provided a measure of knowledge retention and assessed whether improvements in confidence and comfort persisted beyond the immediate post-intervention period.

Additionally, demographic information, like the highest level of education, was collected to examine whether these variables affected instructional methods. Education was grouped as high school diploma or less, currently in college, bachelor’s degree, or postgraduate education. This demographic factor was incorporated into subgroup analyses to determine its potential influence on knowledge gain, retention, and affective outcomes.

Data analysis was conducted using paired t-tests to compare pre- and post-intervention scores within each group, quantifying individual knowledge gains and changes in confidence and comfort. To evaluate differences across groups (poster vs. prosthetic; prostate vs. testicular), independent t-tests were performed. Statistical significance was defined as p < 0.05. These methods ensured robust evaluation of learning gains, behavioral impact, and the relative efficacy of teaching modalities across diverse participant backgrounds.

## Results

**Figure 1:**
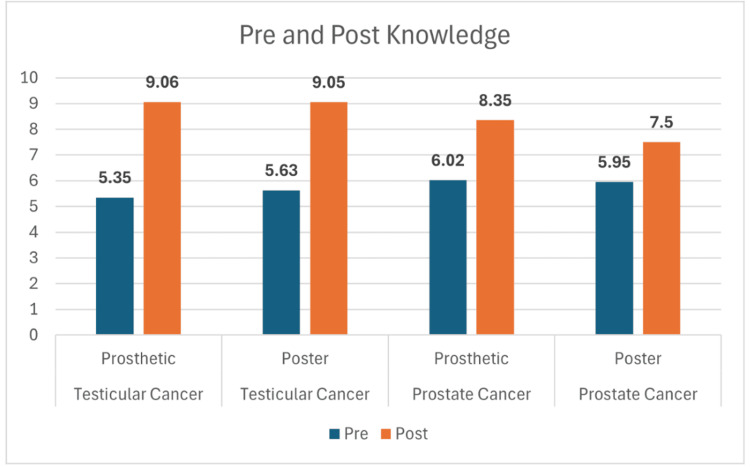
Knowledge change in testicular and prostatic cancer

**Figure 2:**
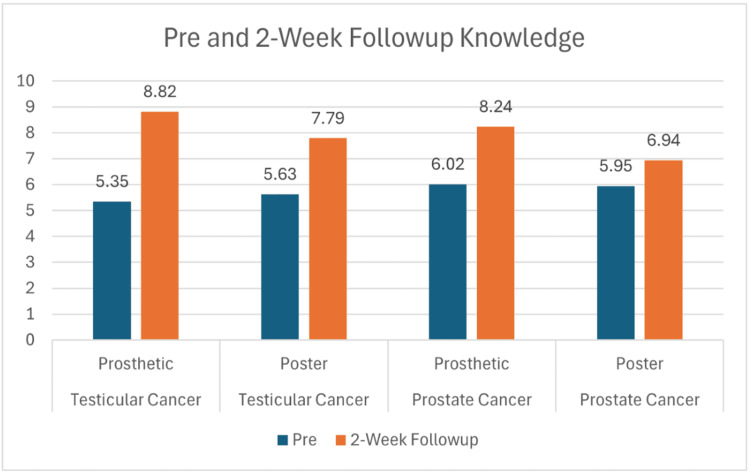
Knowledge retention after two weeks

**Figure 3:**
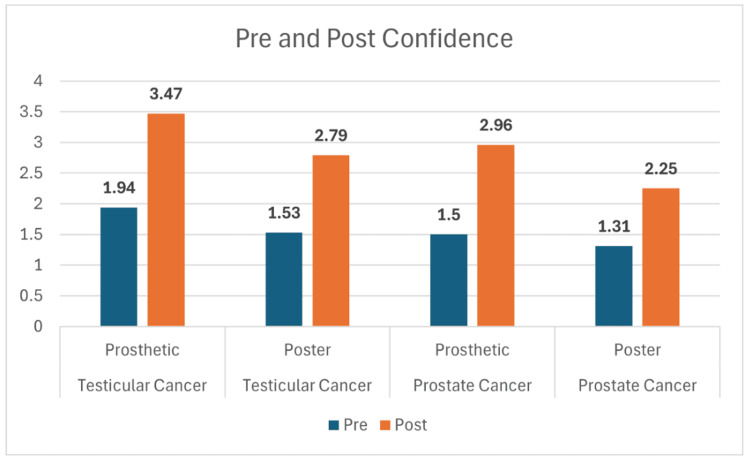
Confidence in self-examination for testicular and prostate cancer

**Figure 4:**
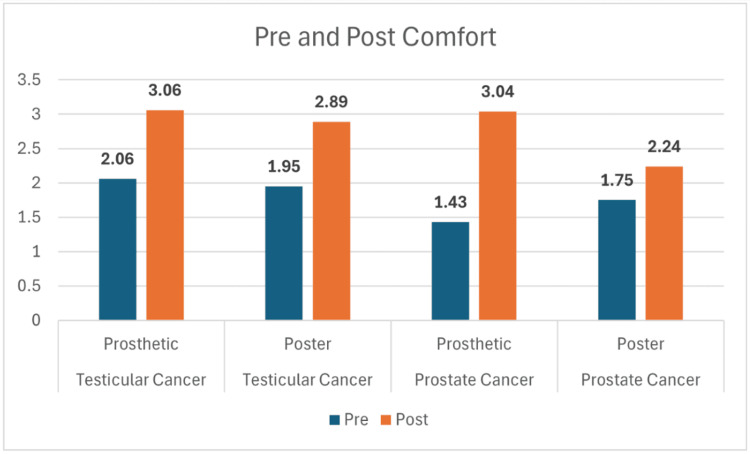
Comfort level in discussion with the physician

Across all four study groups, participants showed marked improvement in knowledge following the educational interventions. For testicular cancer, mean scores increased from 5.35 to 9.06 in the prosthetic group and from 5.63 to 9.05 in the poster group (Figure 1). Similarly, prostate cancer participants improved from 6.02 to 8.35 with the prosthetic and from 5.95 to 7.50 with the poster (Figure 1). While both modalities led to significant learning gains, statistical testing revealed that the prosthetic method produced greater retention at two-week follow-up. Knowledge scores at follow-up remained higher for the prosthetic groups, 8.82 for testicular and 8.24 for prostate, compared with the poster groups at 7.79 and 6.94, respectively (Figure 2). These differences were statistically significant for both cancer types (p = 0.02 for testicular; p = 0.005 for prostate). Both teaching approaches improved participants’ self-reported comfort and confidence in performing self-examinations. For testicular cancer, confidence rose from 1.94 to 3.47 in the prosthetic group and from 1.53 to 2.79 in the poster group (Figure 3). A similar trend was observed in the prostate cancer cohort, where confidence increased from 1.50 to 2.96 with the prosthetic and from 1.31 to 2.25 with the poster (Figure 3). Statistical analysis showed a significant advantage for the prosthetic method in the prostate cancer group (p = 0.006) (Table 1). Comfort scores followed a comparable trend, with prosthetic training again yielding higher post-session gains, particularly for prostate cancer participants, with a mean change of +1.61 vs. +0.69; p = 0.001 (Figure 4, Table 2).

**Table 1:** Confidence in self-examination (pre vs. post)

Cancer type	Tool	Mean change	t-statistic	p-value	Significant?
Testicular	Prosthetic vs. poster	+1.53 vs. +1.26	1.78	0.08	No
Prostate	Prosthetic vs. poster	+1.46 vs. +0.94	2.91	0.006	Yes

**Table 2:** Comfort level (pre vs. post)

Cancer type	Tool	Mean change	t-statistic	p-value	Significant?
Testicular	Prosthetic vs. poster	+1.00 vs. +0.95	0.28	0.78	No
Prostate	Prosthetic vs. poster	+1.61 vs. +0.69	3.45	0.001	Yes

## Discussion

Participants who engaged with prosthetic models demonstrated greater immediate knowledge gains compared to those who viewed poster-based materials across both testicular and prostate cancer modules. This finding reinforces the benefits of multimodal instructional design, where tactile and visual inputs complement information learning to facilitate deeper conceptual understanding [9].

Hands-on interaction enables participants to internalize anatomical structures, palpation techniques, and cancer warning signs more effectively than passive observation alone. These results align with evidence that experiential learning supports more robust knowledge acquisition in health education settings, particularly for procedural and anatomy-focused content [10]. Such improvement is clinically meaningful given that knowledge deficits remain a major barrier to early urologic cancer detection in the general population. By enhancing foundational understanding, prosthetic-based instruction may support earlier recognition of concerning symptoms and promote evidence-based screening behaviors [11]. Participants with lower education, especially those with only a high school diploma, exhibited the largest improvements in both knowledge and confidence from prosthetic-based instruction, with bachelor’s and postgraduate groups also benefiting more from prosthetics than posters (Table 3). These findings are consistent with evidence from simulation-based medical education, which has shown that interactive, tactile models enhance comprehension and retention by engaging multiple sensory pathways, reinforcing anatomical relationships, and improving procedural understanding [12].

**Table 3:** Comparison of knowledge gains after cancer education by education level and teaching modality

Cancer type	Teaching modality	Knowledge gained in high school	Knowledge gained in a bachelor’s degree program	Knowledge gained in a master’s or higher degree program
Testicular	Prosthetic	3.81	3.52	3.47
	Poster	3.31	3.25	3.31
Prostate	Prosthetic	2.96	2.88	2.82
	Poster	1.92	2.43	2.38

At a two-week follow-up, participants in the prosthetic-based learning groups retained a higher proportion of their initial knowledge gains compared with those exposed only to posters. Although all groups demonstrated some decline, the sustained performance among prosthetic users suggests superior long-term consolidation. Learning science literature attributes this phenomenon to multisensory reinforcement and active learning, both of which promote long-term memory [13]. Notably, the most pronounced retention advantages were seen among participants with lower baseline education levels. This supports that tactile educational modalities may play a role in mitigating disparities in cancer literacy by offering accessible, concrete learning experiences [14]. These data highlight the potential value of incorporating physical models into public health cancer education campaigns, particularly in populations at risk of limited knowledge durability.

Prosthetic-based teaching produced greater improvements in self-reported comfort initiating cancer-related discussions with healthcare providers compared to poster-based instruction. This effect was especially prominent in the prostate cancer cohort, where stigma, embarrassment, and uncertainty frequently impede communication about genitourinary concerns [15]. Physical models may serve to normalize anatomical dialogue, reduce discomfort associated with sensitive health topics, and create psychologically safe learning environments that encourage engagement. These findings support prior work demonstrating that interactive, simulation-based cancer education can reduce emotional barriers, enhance readiness to seek help, and improve willingness to disclose symptoms [16]. Improving communication comfort is critical given that patient-clinician dialogue represents a pivotal step in early cancer detection and timely diagnostic evaluation.

Participants trained with prosthetic models also reported greater increases in confidence performing testicular and prostate self-examination techniques. This can be supported by the self-efficacy theory, which suggests that hands-on skill rehearsal is fundamental to developing procedural confidence [3]. Prosthetic models allow learners to familiarize themselves with anatomical landmarks, practice palpation strategies, and internalize tactile cues in a controlled environment without fear of error or judgment [17]. Conversely, poster-based instruction, while informative, does not facilitate motor learning or haptic skill acquisition. Given that confidence strongly predicts adoption and maintenance of self-screening behaviors, the observed improvements may have direct implications for real-world practice [18].

Although the testicular cancer results in this study did not reach statistical significance, the data still demonstrated meaningful positive trends in participant outcomes. The small sample size for the testicular cancer subset (n = 36) likely contributed to these differences not achieving statistical significance, despite consistent directional improvement across all measured domains (Table 1, Table 2).

Future application

The enhanced outcomes associated with prosthetic models suggest that tactile, interactive tools may be especially effective in cancer education. Future efforts could include the development of prosthetic-based teaching tools in primary care settings, schools, and even community outreach events. Additionally, with the increasing accessibility of virtual reality and 3D simulation, future studies could compare these with physical prosthetics and posters to assess cost-effectiveness and reach.

Limitations

The relatively small sample size may limit the statistical power and generalizability of the findings. Participants were likely recruited from a limited geographic or institutional pool, which may not reflect broader population demographics. Additionally, comfort and confidence levels were based on self-reported measures, which can be subject to response bias. Participants may have over- or under-reported their confidence or knowledge levels.

Knowledge retention was only assessed at a two-week follow-up, limiting insight into long-term educational efficacy; longer-term follow-up (e.g., three to six months) would better evaluate sustained impact. Additionally, although knowledge and self-reported comfort were measured, no behavioral outcomes were tracked, such as whether participants ultimately performed self-examinations or discussed screening with a physician. Feasibility and cost considerations were not assessed, which may affect real-world implementation, especially in settings with limited instructional time, staffing, or educational resources. Resource constraints, such as access to trained educators, teaching materials, or digital platforms, may impede scalability. Cultural and linguistic differences were also not evaluated, and educational materials may not equally resonate across diverse communities, creating potential gaps in applicability and engagement. Finally, participants with prior exposure to cancer-related topics may have had differing baseline knowledge or motivation, introducing familiarity bias that could have influenced intervention effects.

## Conclusions

This study demonstrates that prosthetic-based education is a more effective teaching tool than poster-based methods for improving knowledge, retention, confidence, and comfort regarding prostate and testicular cancer. By offering tactile, interactive engagement, prosthetic models reinforce anatomical understanding and procedural skills in ways that visual-only materials cannot. These benefits were especially pronounced among individuals with lower educational attainment and older adults, highlighting their potential to reduce disparities in health literacy and strengthen cancer prevention efforts.

Integrating prosthetic models into public health education programs may therefore enhance cancer awareness, foster long-term adoption of self-examination practices, and reduce the stigma that often surrounds male reproductive health. By equipping men with the knowledge and confidence to engage in early detection behaviors and to communicate openly with healthcare providers, such interventions hold promise for earlier diagnosis and ultimately lower mortality rates associated with prostate and testicular cancers.

## Appendices

**Table 4:** Prostate Cancer Questionnaire

Question	Choice 1	Choice 2	Choice 3	Choice 4	Choice 5	Choice 6
1. Age (pick one)	Under 20	20–29	30–39	40–49	50 and above	–
2. Highest degree or level of education completed (pick one)	Did not finish high school	High school diploma	Bachelor’s degree / In college	Master’s or higher	–	–
3. Have you ever discussed prostate cancer or prostate self-exams with a healthcare professional? (pick one)	Yes	No	–	–	–	–
4. Have you or anyone you know been diagnosed with prostate cancer? (pick one)	Yes	No	–	–	–	–
5. Are you currently working in the healthcare field? (pick one)	Yes	No	–	–	–	–
6. What fraction of males will get prostate cancer? (pick one)	1 in 8	1 in 20	1 in 100	1 in 250	1 in 1000	–
7. How confident are you in performing a prostate cancer self-check? (pick one)	Very confident	Somewhat confident	Not very confident	Not at all confident	–	–
8. Which of the following are risk factors? (pick all that apply)	Family history	Environmental exposures	Smoking	Diet/lifestyle	Age	Race/Ethnicity / None
9. Signs & symptoms of prostate cancer (pick all that apply)	Difficulty urinating	Erectile dysfunction	Blood in urine or semen	Burning with urination	Sexual activity increases risk*	–
10. Treatment options for prostate cancer (pick all that apply)	Active surveillance	Radical prostatectomy	Radiation therapy	Herbal supplements	Hormone therapy	Bone marrow transplant / Lifestyle changes
11. Can treatment affect fertility or sexual function? (pick one)	Yes	No	Unsure	–	–	–
12. Comfort discussing prostate health with provider (pick one)	Very comfortable	Somewhat comfortable	Not very comfortable	Not at all comfortable	–	–

**Table 5:** Testicular Cancer Questionnaire

Question	Choice 1	Choice 2	Choice 3	Choice 4	Choice 5	Choice 6
1. Age (pick one)	Under 20	20–29	30–39	40–49	50 and above	–
2. Highest degree or education level completed (pick one)	Did not finish high school	High school diploma	Bachelor’s degree / in college	Master’s or higher	–	–
3. Have you ever discussed testicular cancer or TSE with a healthcare provider? (pick one)	Yes	No	–	–	–	–
4. Have you or anyone you know been diagnosed with testicular cancer? (pick one)	Yes	No	–	–	–	–
5. Are you currently working in the healthcare field? (pick one)	Yes	No	–	–	–	–
6. What fraction of males will get testicular cancer? (pick one)	1 in 100	1 in 250	1 in 1000	1 in 2500	1 in 5000	–
7. How confident are you in your ability to perform a testicular cancer self-check? (pick one)	Very confident	Somewhat confident	Not very confident	Not at all confident	–	–
8. Which of the following are risk factors? (pick all that apply)	Family history	Undescended testicle	Previous testicular cancer	Abnormal testicular development	Age	Race/Ethnicity
9. What are the signs/symptoms of testicular cancer? (pick all that apply)	Symptoms limited to the testes	Painless lump in testicle/scrotum	Heaviness in the scrotum	Most have visible large lumps	Usually affects one testicle	Without a family history, the risk is negligible
10. Treatment options for testicular cancer (pick all that apply)	Hormone therapy	Radiation therapy	Orchiectomy	Chemotherapy	Active surveillance	–
11. Can treatment affect fertility or sexual function? (pick one)	Yes	No	Unsure	–	–	–
12. Comfort discussing testicular health with a provider (pick 1)	Very comfortable	Somewhat comfortable	Not very comfortable	Not at all comfortable	–	–
